# Beyond self-reports: serum cotinine reveals sex-and age-related differences of smoking on all-cause and disease-specific mortality

**DOI:** 10.3389/fpubh.2025.1512603

**Published:** 2025-02-17

**Authors:** Qi Jiang, Liu Junjun, Xiaochuan Wang, Li Luo, Gaoyan He, Xiaojuan Wu, Qian Min, Ying Long, Wang Wenjun, Tao Zhu, Yu Yao

**Affiliations:** ^1^Department of Pediatrics, Suining Central Hospital, Suining, China; ^2^Department of Respiratory and Critical Care Medicine, The Affiliated Hospital, Southwest Medical University, Luzhou, China; ^3^Department of Respiratory Medicine and Critical Care Medicine, Suining Central Hospital, Suining, China

**Keywords:** cotinine, cigarette exposure, all-cause mortality, cardiovascular disease, cancer mortality, threshold saturation effects transferase, ion selective electrode, least absolute shrinkage and selection operator

## Abstract

**Background:**

It is well-known that sex and age play critical roles in smoking-related diseases and mortality. However, quantification of the extent of smoking requires self-reports in these studies, which may yield only partially accurate results. This study investigated sex-and age-related differences in the association between smoking and all-cause, cardiovascular disease, and cancer mortality by measuring serum cotinine levels.

**Methods:**

Participants aged 20–85 years from the US National Health and Nutrition Examination Survey (1999–2018) were included. All-cause and disease-specific mortality data were obtained from publicly available user-linked mortality files. Multivariate Cox regression was performed to identify serum cotinine as an independent risk factor of mortality. Subgroup and interaction analyses were performed to investigate these sex and age differences. Smooth curve fitting was conducted to discover potential nonlinear relationships and threshold saturation effects.

**Results:**

Sex was significantly associated with all-cause and cancer mortality. Threshold saturation effects were observed in all-cause mortality among both males and females, cancer mortality among females, and cardiovascular disease mortality among males. Age markedly associated with all-cause and cardiovascular disease mortality. Threshold saturation effects were found in cardiovascular disease mortality among younger adults and cancer mortality among the all-age population.

**Conclusion:**

These findings suggest that there are threshold saturation effects between smoking and mortality, and sex and age differences in smoking-related mortality are inconsistent in different diseases.

## 1 Introduction

Despite annualized rates of decline in smoking prevalence, smoking remains the second leading risk factor for early death and disability worldwide, contributing to 11.5% of global deaths (6.4 million), of which more than half occur in four countries (1).

Evidence from several extensive contemporary studies illustrates sex and age differences in the health outcomes of tobacco exposure, suggesting that the comprehensive risk of smoking is considerably more significant in women than in men, and in young adults than in older adults (2–8). For example, in 2014, a cohort study with an extended follow-up indicated that the full hazards of light smoking (1–14 cigarettes per day) at baseline were more significant for women than for men, while the effect decreased with increasing extent of smoking, suggesting a dose–response relationship (9). The risks of mortality and health outcomes vary according to the age at smoking initiation. A prospective cohort study including a contemporary US population showed that delayed smoking initiation reduced the mortality risk of cancer (7).

A limitation in these studies is that the quantification of the extent of smoking was subjective and self-reported, including the pack-year index and daily cigarette consumption. Self-reports are frequently affected by memory and emotion-expression biases (10). Meanwhile, the adverse health impact of actual exposure to smoke toxicants is determined by multiple factors, including the number of smoked cigarettes, smoking years, cigarette brands, cigarette emissions, and even smoking topography (11–14). Therefore, cigarette consumption may not fully reflect the extent of the harmful effects of smoking. Moreover, it is complicated to quantify the extent of passive smoking by the frequency of smoking and the number of cigarettes consumed. The methods of tobacco exposure quantification may have resulted in inaccuracies in these previous studies regarding the sex and age differences in smoking. Hence, an objectively quantifiable indicator is required to evaluate these differences in smoking.

Cotinine is the most predominant metabolite of nicotine, which is similarly a tobacco-specific biomarker (15–17). Serum cotinine levels measured at a single time point are positively associated with the degree of tobacco smoke exposure, considered one of the most reliable indicators of both smoking intensity and exposure to environmental tobacco smoke, which includes first-hand, second-hand, and third-hand smoke exposure (18, 19). Meanwhile, cotinine-assessed smoking status is generally more accurate than self-reported smoking status (20). Smoking is associated with mortality from various diseases, including cardiovascular diseases and cancer (1). A National Health and Nutrition Examination Survey (NHANES) of 20,175 self-reported non-smokers aged ≥20 years revealed that serum cotinine levels were significantly associated with death from lung cancer, all cancers, and heart diseases (21).

These findings support the assertion that cotinine is an effective indicator to evaluate smoking-related mortality risk. Another related question arises from these results, namely, whether there are thresholds and differences among different diseases in sex-and age-related differences in smoking. In particular, there is a need for long-term prospective studies to completely confirm these differences by measuring serum cotinine with standard statistical methods. Therefore, this study aimed to investigate sex-and age-related differences in smoking-related mortality by measuring serum cotinine levels, emphasizing subgroup analyses and possible nonlinear relationships and threshold saturation effects.

## 2 Materials and methods

### 2.1 Study design

This prospective cohort study utilized data from 10 cycles (1999 and 2018) of the US NHANES (RRID:SCR_013201). The NHANES is a series of ongoing cross-sectional surveys with a nationally representative sample of non-institutionalized individuals conducted by the National Center for Health Statistics (NCHS) at the US Center for Disease Control. It includes data from health survey interviews, physical examinations, questionnaires, laboratory, and mortality. All-cause and disease-specific mortality data from 1999 to 2018 were obtained from publicly available-user-linked mortality files, linked to the National Death Index. All participants provided written consent, and the NCHS Ethics Review Board approved this study (Protocols #98–12, #2005–06, #2011–17, and #2018–01).

### 2.2 Participants

Participants aged 20–85 years in the NHANES (1999–2018) with serum cotinine and follow-up data were included in this study. The exclusion criteria were (1) missing data; (2) positive test results for tuberculosis (TB); (3) human immunodeficiency virus (HIV) antibody; (4) hepatitis C antibody; (5) hepatitis B surface antibody; (6) ever had chronic obstructive pulmonary disease; (7) ever had and still have a liver condition; (8) ever had a stroke, heart attack, angina/angina pectoris, coronary heart disease, congestive heart failure; (9) ever received blood transfusions; (10) ever had attention deficit disorder; (11) received treatment for anemia in/the past three months.

### 2.3 Variables

#### 2.3.1 Serum cotinine

Serum cotinine concentration was measured using an isotope-dilution high-performance liquid chromatography/atmospheric pressure chemical ionization tandem mass spectrometric method.

##### 2.3.1.1 Routine biochemistry profiles and complete blood count (CBC)

In the NHANES 2005–2007, 2008–2010/2015–2016, and 2017–2018, routine biochemical profiles and CBC were analyzed using Beckman Synchron LX20 Beckman DxC800 (Beckman Coulter, Fullerton, CA, US), and Roche Cobas 6,000 (c501 module) (Cobas 6,000; Roche Diagnostics USA), respectively were used. Further details of this analysis are in the Supplementary material.

BMI, smoking status, family income to poverty ratio, blood pressure (BP), glycohemoglobin, high-density lipoprotein cholesterol (HDL-C), and total nutrient intake.

Obesity was defined as BMI ≥30. Smoking status was defined as SHS (not actively smoking but exposed to tobacco smoke in the workplace or at home in the past 7 days); non-smoking (smoking <100 cigarettes during the lifetime, not smoking currently, and not exposed to environmental tobacco smoke); and active smoking (smoked >100 cigarettes during the lifetime and currently smoking cigarettes). The family income to poverty ratio was calculated by comparing family (or individual) income to the poverty guidelines specific to the survey year.

##### 2.3.1.2 Blood pressure (BP)

BP was measured by Shared Care Research and Education Consulting trained investigators, certified to measure the BP.

##### 2.3.1.3 HDL-C and glycohemoglobin

HDL-C and glycohemoglobin were measured using Cobas 6,000 Chemistry Analyzer and Tosoh G8 Glycohemoglobin Analyzer, respectively.

##### 2.3.1.4 Total nutrient intakes

Total nutrient intakes were used to estimate the types and amounts of foods and beverages (including all types of water) consumed during the 24-h period prior to the interview (midnight to midnight) conducted at mobile examination centers, and to estimate the intake of energy, nutrients, and other food components from those foods and beverages.

### 2.4 Statistical analyses

Normally distributed data was presented by mean ± standard deviation (SD). Categorical variables were analyzed by Chi-square test. Continuous variables with normal distribution were analyzed by Student t test. Ordinal variables and continuous variables without normal distribution were analyzed by Mann–Whitney U-test. Variance inflation factor (VIF) was performed to analyze data collinearity. Least absolute shrinkage and selection operator (LASSO) was used to select potential variables associated with all-cause, cardiovascular disease, and cancer mortality. Subsequently, based on the variables selected by LASSO regression, three multivariate Cox regression models were established to identify the independent risk factors for mortality. Prior to applying the Cox proportional hazards model, a time-dependent model was employed to verify the proportionality of risk for the aforementioned factors, all of which satisfied the equal proportionality hypothesis. The potential effect modifications of sex (male vs. female) and age (<65 years vs. ≥65 years) were evaluated using subgroup and interaction analyses. Smooth curve fitting was performed to examine whether serum cotinine levels were partitioned into intervals, and segmented regression. Log-likelihood ratio tests were performed to determine whether a threshold existed in the association between serum cotinine levels and mortality. These analyses were conducted using both logarithmic transformed and untransformed data. The log (relative risk) was converted to a relative risk by taking the antilog. Nomograms and receiver operating characteristic (ROC) curves were used to visualize and verify the three multivariate Cox regression models. The R project (http://www.Rproject.Org/; RRID:SCR_001905) and Empower Stats (http://www.Empowerstats.com) were used for all statistical analyses. We utilized the MEC subsample weights, specifically WTMEC4YR for the years 1999–2002 and WTMEC2YR for 2003–2018, along with the masked variance unit Pseudo-PSU variable (SDMVPSU) and the masked variance unit Pseudo-Stratum variable (SDMVSTRA) for variance estimation in this analysis. These sampling weights were recalculated to account for the combination of 10 NHANES cycles. Based on the NCHS edited analytical guidelines. Statistical significance was set at *p* < 0.05.

## 3 Results

### 3.1 Baseline characteristics of participants by sex

**Figure 1:**
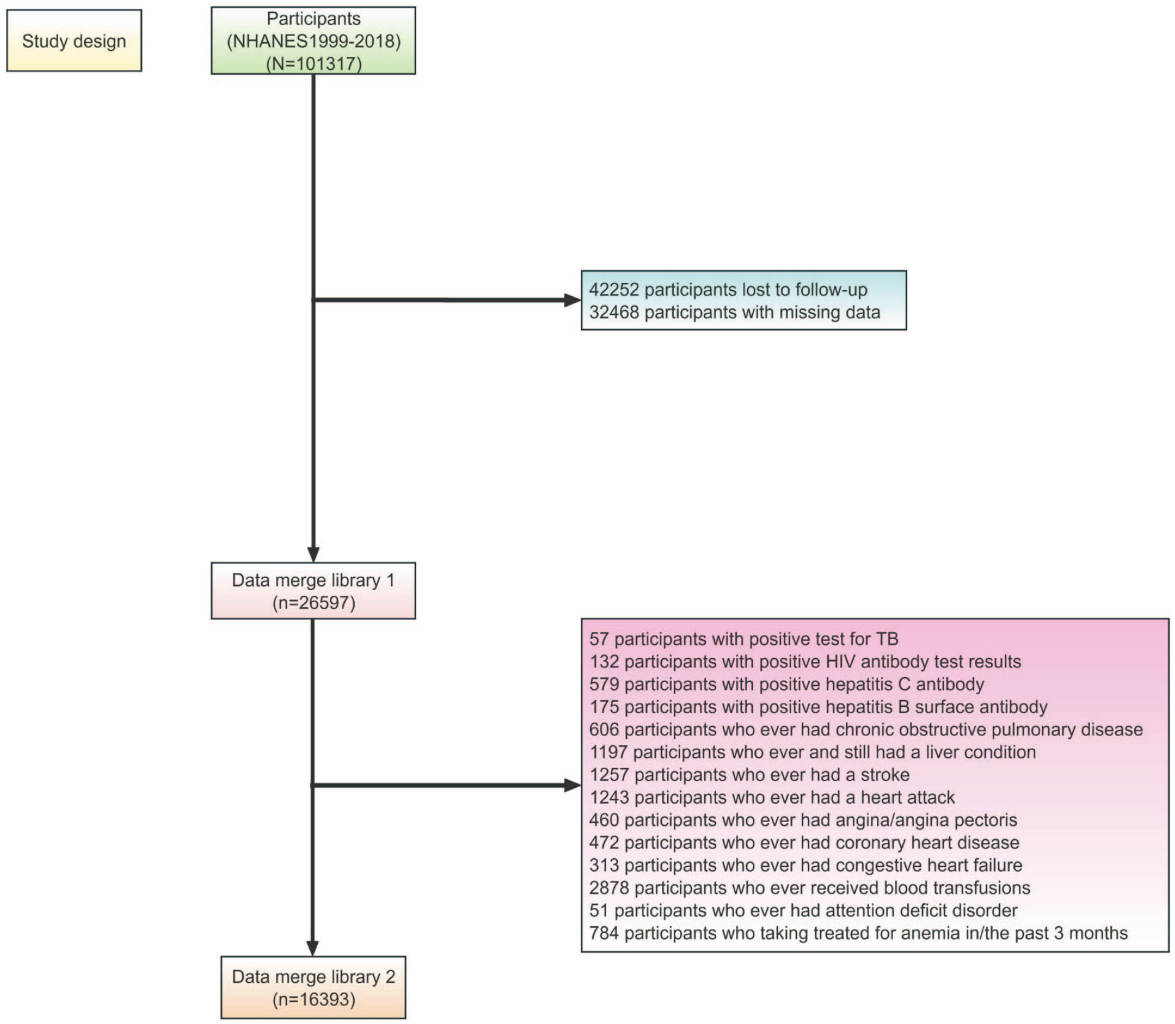
Summary of study design.

In total, 16,393 participants (male 52.94%; Figure 1) were included in the study. The baseline characteristics of the study participants are presented in Table 1 and Supplementary Table S1. In both groups, all baseline characteristics differed significantly. Of these, the males had significantly higher serum cotinine levels and higher all-cause, cardiovascular disease, and cancer mortality than the females.

**Table 1:** Baseline characteristics of participants, NHANES 1999–2018 (*N* = 16,393).

Variables	Males (*n* = 8,678)	Females (*n* = 7,715)	P
Age (year)	46.61 ± 17.04	45.71 ± 16.83	<0.001
Ratio of family income to poverty	2.62 ± 1.62	2.53 ± 1.63	<0.001
Cotinine, serum (ng/ml)	90.90 ± 152.85	59.90 ± 123.03	<0.001
Systolic blood pressure (SBP) (mm Hg)	125.61 ± 16.56	120.90 ± 18.94	<0.001
Diastolic blood pressure (DBP) (mm Hg)	72.21 ± 12.70	69.19 ± 12.36	<0.001
Body mass index (BMI) (kg/m2)	28.47 ± 5.95	29.26 ± 7.39	<0.001
Race (*n*, %)			<0.001
Mexican American	1,467 (16.90%)	1,149 (14.89%)	<0.001
Other Hispanic	697 (8.03%)	666 (8.63%)	<0.001
Non-Hispanic White	3,864 (44.53%)	3,596 (46.61%)	<0.001
Non-Hispanic Black	1725 (19.88%)	1,476 (19.13%)	<0.001
Other Race - Including Multi-Racial	925 (10.66%)	828 (10.73%)	<0.001
Education level (*n*, %)			<0.001
Less than 9th grade	899 (10.36%)	582 (7.54%)	<0.001
9-11th grade (Includes 12th grade with no diploma)	1,274 (14.68%)	982 (12.73%)	<0.001
High school graduate/GED or equivalent	2,191 (25.25%)	1733 (22.46%)	<0.001
Some college or AA degree	2,388 (27.52%)	2,614 (33.88%)	<0.001
College graduate or above	1923 (22.16%)	1803 (23.37%)	<0.001
Do not know	3 (0.03%)	1 (0.01%)	<0.001
Smoking status (*n*, %)			
Non-smoker	4,743 (54.66%)	4,608 (59.73%)	<0.001
Active smoker	2,183 (25.16%)	1,632 (21.15%)	<0.001
Second-hand smoker	1752 (20.19%)	1,475 (19.12%)	<0.001
Cancer or malignancy history (*n*, %)	524 (6.04%)	601 (7.79%)	<0.001
All-cause mortality (*n*, %)	780 (8.99%)	451 (5.85%)	<0.001
Cardiovascular disease mortality (*n*, %)	190 (2.19%)	95 (1.23%)	<0.001
Cancer mortality (*n*, %)	223 (2.57%)	116 (1.50%)	<0.001
Months of Follow-up (month)	94.79 ± 55.97	91.89 ± 57.16	<0.001

### 3.2 LASSO regression analysis of potential variables

LASSO was used to reduce the dimensions of the data and select the potential variables of all-cause, cardiovascular disease, and cancer mortality (Supplementary Figure S1). We identified 22, 15, and 8 variables, respectively (Table 2).

**Table 2:** LASSO regression analysis of potential variables associated with mortality.

	All-cause mortality (*n* = 16,393)	Cardiovascular disease mortality (*n* = 15,448)	Cancer mortality (*n* = 15,502)
Variables	Coefficient	Coefficient	Coefficient
Serum cotinine	0.000764483309013736	0.000560233200094427	0.000996556393966816
Albumin	−0.0229294791491493	−0.0277828960058909	−1.63419640188636 × 105
ALP	0.00147120337374417	0.00277268892675989	-
BUN	0.0249792853890213	0.07849728135601	-
Globulin	0.0152258065184912	0.000851504205504739	-
Serum glucose	0.0332404888808222	-	-
GGT	0.000131516508822317	-	-
LDH	0.000889730084623608	0.00296655343332179	-
Potassium	0.0767125924201666	-	-
UA	0.000243516686654068	0.000477059666618895	-
SBP	0.00436208477508385	0.0096612766809135	-
Monocyte number	-	0.0581686128968135	-
Neutrophils number	0.0221959188944507	-	0.0108213314711999
MCV	0.0112758351650363	-	-
RDW	0.159243674395514	0.103082670123705	0.064822657893425
Male	0.133285196248189	0.175410048267093	0.0334566378759417
Female	−0.00536932320296478	0.175410048267093	−3.89494553060686 × 1013
Age	0.0723914575297077	0.0807970343986578	0.0778413287043587
Mexican American	−0.0070064505814015	-	-
Non-Hispanic White	0.0913340491033871	-	-
Ratio of family income to poverty	−0.0719216600548048	−0.0935663383494493	-
Glycohemoglobin	0.00613853610261398	0.0205056514177715	-
Cancer or malignancy history	0.11932239367294	-	0.447915763423953

### 3.3 Multivariate cox regression analysis

Three multivariate Cox regression analysis models, including the variables selected using LASSO, were established (Table 3). After adjusting for Albumin, ALP, BUN, globulin, serum glucose, GGT, LDH, potassium, uric acid, SBP, neutrophils number, MCV, RDW, gender, age, race, the ratio of family income to poverty, glycohemoglobin, and cancer or malignancy history, after adjusting for Albumin, ALP, BUN, globulin, LDH, uric acid, SBP, monocyte number, RDW, gender, age, the ratio of family income to poverty, and glycohemoglobin, after adjusting for Albumin, ALP, neutrophils number, RDW, gender, age, and cancer or malignancy history, serum cotinine levels were found to be positively associated with all-cause, cardiovascular disease, and cancer mortality, respectively.

**Table 3:** Multivariate cox regression analysis of independent variables associated with mortality.

	All-cause mortality		Cardiovascular disease mortality		Cancer mortality	
Variables	HR95% CI	P	HR95% CI	P	HR95% CI	P
Serum cotinine	1.0012 (1.0007, 1.0016)	<0.0001	1.0013 (1.0006, 1.0021)	0.0004	1.0021 (1.0014, 1.0028)	<0.0001
Albumin	0.9532 (0.9322, 0.9748)	<0.0001	0.9299 (0.8904, 0.9711)	0.001	0.9434 (0.9000, 0.9888)	0.0152
ALP	1.0027 (0.9995, 1.0060)	0.1016	1.0056 (0.9993, 1.0119)	0.0792	-	-
BUN	1.0348 (0.9968, 1.0742)	0.0729	1.0892 (1.0264, 1.1559)	0.0048	-	-
Globulin	1.0378 (1.0229, 1.0529)	<0.0001	1.0375 (1.0020, 1.0744)	0.0383	-	-
Serum glucose	1.0379 (0.9870, 1.0914)	0.1470	-	-	-	-
GGT	1.0007 (0.9997, 1.0042)	0.1813	-	-	-	-
LDH	1.0023 (1.0004, 1.0042)	0.0181	1.0053 (1.0035, 1.0072)	<0.0001	-	-
Potassium	1.3034 (1.0840, 1.5672)	0.0048			-	-
Uric acid	1.0006 (0.9996, 1.0016)	0.2490	1.0017 (0.9993, 1.0042)	0.1627	-	-
SBP	1.0061 (1.0032, 1.0090)	<0.0001	1.0123 (1.0055, 1.0191)	0.0004	-	-
Monocyte number	-		1.3714 (0.9414, 1.9979)	0.0999	-	-
Neutrophils number	1.0437 (1.0186, 1.0695)	0.0006	-	-	1.0825 (1.0454, 1.1210)	<0.0001
MCV	1.0380 (1.0208, 1.0554)	<0.0001	-	-	-	-
RDW	1.2674 (1.1945, 1.3448)	<0.0001	1.2096 (1.0946, 1.3367)	0.0002	1.2507 (1.1406, 1.3716)	<0.0001
Female	0.6950 (0.5950, 0.8119)	<0.0001	0.4521 (0.3160, 0.6470)	<0.0001	0.6569 (0.5112, 0.8440)	0.001
Age	1.0752 (1.0672, 1.0833)	<0.0001	1.1016 (1.0849, 1.1186)	<0.0001	1.1035 (1.0911, 1.1160)	<0.0001
Mexican American	0.7268 (0.5689, 0.9286)	0.0107	-	-	-	-
Non-Hispanic White	1.2028 (1.0268, 1.4091)	0.0222	-	-	-	-
Ratio of family income to poverty	0.8686 (0.8279, 0.9113)	0.0034	0.8647 (0.7703, 0.9707)	0.0137	-	-
Glycohemoglobin	1.0675 (0.9811, 1.1813)	0.0006	1.1372 (0.9921, 1.3035)	0.0649	-	-
Cancer or malignancy history	0.7538 (0.6241, 0.9105)	<0.0001	-	-	0.5364 (0.3922, 0.7334)	<0.0001

### 3.4 Subgroup and interaction analyses

The subgroup and interaction analyses of the association between serum cotinine levels and mortality stratified by various causes are shown in Table 4 after adjusting the above covariates. The positive role of serum cotinine level in increasing mortality was significant in most subgroups. However, the positive associations of all-cause and cardiovascular disease mortality with serum cotinine levels were not significant in older adults. The positive association between cardiovascular disease mortality and serum cotinine levels remained insignificant in the participants with obesity. In the interaction analyses, the hazard ratio (HR) among females was greater than that for males for all mortality outcomes, suggesting that serum cotinine levels have a higher mortality risk in females. Moreover, we found that sex interacted with serum cotinine levels for all-cause and cancer mortality, and age (<65 years vs. ≥65 years) interacted with serum cotinine levels for all-cause and cardiovascular disease mortality.

**Table 4:** Subgroup and interaction analyses.

Subgroups	N	HR95% CI	P	Interaction *P*
All-cause mortality				
Male	8,678	1.0006 (1.0001, 1.0011)	0.0187	0.0173
Female	7,715	1.0021 (1.0015, 1.0027)	<0.0001	
<65 years	13,598	1.0012 (1.0006, 1.0017)	<0.0001	0.0083
≧65 years	2,795	1.0005 (0.9999, 1.0011)	0.1335	
Non-obese	10,431	1.0010 (1.0005, 1.0015)	<0.0001	
Obese	5,962	1.0012 (1.0006, 1.0018)	0.0001	
Cardiovascular disease mortality				
Male	8,088	1.0011 (1.0002, 1.0020)	0.0136	0.0520
Female	7,359	1.0026 (1.0014, 1.0038)	<0.0001	
<65 years	13,234	1.0023 (1.0013, 1.0032)	<0.0001	0.0123
≧65 years	2,213	1.0002 (0.9989, 1.0015)	0.7254	
Non-obese	9,801	1.0017 (1.0009, 1.0026)	<0.0001	
Obese	5,646	1.0012 (1.0000, 1.0024)	0.0587	
Cancer mortality				
Male	8,121	1.0014 (1.0007, 1.0021)	<0.0001	0.0294
Female	7,380	1.0031 (1.0021, 1.0041)	<0.0001	
<65 years	13,276	1.0019 (1.0011, 1.0028)	<0.0001	0.4550
≧65 years	2,225	1.0014 (1.0005, 1.0023)	0.0029	
Non-obese	9,840	1.0020 (1.0013, 1.0026)	<0.0001	
Obese	5,661	1.0017 (1.0006, 1.0027)	0.0018	

### 3.5 Segmented regression and log-likelihood ratio test

**Figure 2:**
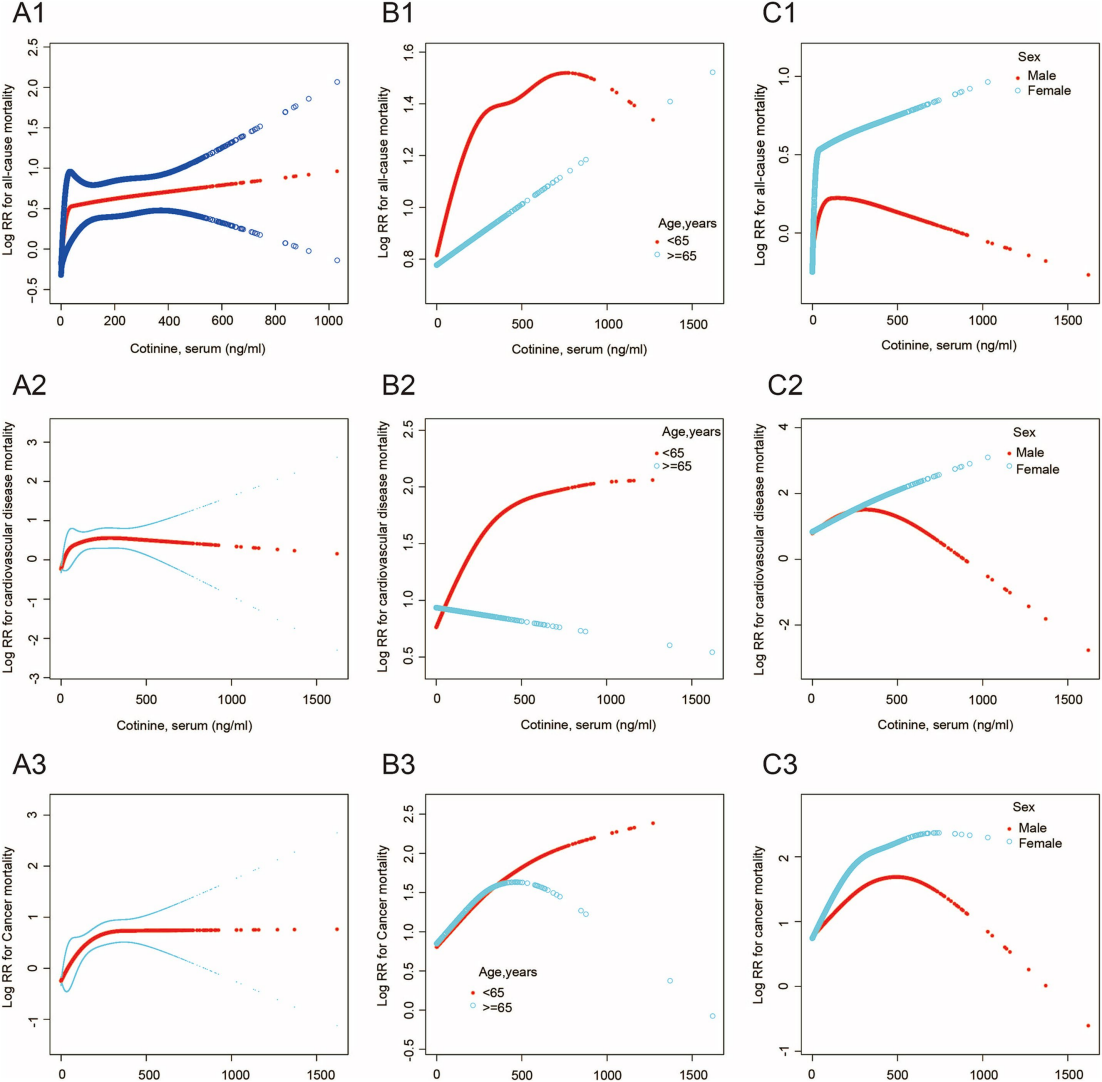
The association between serum cotinine level and mortality. The blue and black lines indicate 95% CI, and the red lines indicate a smooth curve fitting line. **(A1–A3)** The smooth curve of the relationship between serum cotinine level and all-cause, cardiovascular disease, and cancer mortality is shown. **(B1–B3)** The smooth curve of the relationship between serum cotinine level and the above mortality in different age groups is shown. **(C1–C3)** The smooth curve of the relationship between serum cotinine level and the above mortality in different sex groups is shown.

Additive Cox proportional models (Figure 2) were used to visually assess the functional relationship between serum cotinine and all mortality outcomes after adjusting the above covariates. Our data showed that the relationships between serum cotinine levels and mortality outcomes were nonlinear and showed threshold saturation effects (Supplementary Figures S2A1–A3). As shown in Table 5, the cutoffs of the associations of serum cotinine levels with all-cause, cardiovascular disease, and cancer mortality were 11.6, 32.1, and 307 ng/mL, respectively. On the left side of the cutoff values, the risks of these mortality outcomes increased with increasing serum cotinine levels. On the right side of the cutoffs, their risks did not significantly increase with the serum cotinine levels.

**Table 5:** Association between serum cotinine and mortality by segmented regression and log-likelihood ratio test.

	Cutoff	HR95% CI	P
All-cause mortality	< 11.6 ng/mL	1.046 (1.029, 1.063)	<0.0001
	≧11.6 ng/mL	1.000 (0.999, 1.001)	0.8118
Male	< 12.1 ng/mL	1.033 (1.013, 1.054)	0.0010
	≧12.1 ng/mL	1.000 (0.999, 1.000)	0.5146
Female	<36.4 ng/mL	1.023 (1.013, 1.032)	<0.0001
	≧36.4 ng/mL	1.000 (0.999, 1.001)	0.5138
Cardiovascular disease mortality	<32.1 ng/mL	1.022 (1.010, 1.034)	0.0003
	≧32.1 ng/mL	1.000 (0.999, 1.001)	0.7962
Male	<334 ng/mL	1.003 (1.001, 1.004)	<0.0001
	≧334 ng/mL	0.996 (0.992, 1.001)	0.0929
<65 years	<288 ng/mL	1.004 (1.003, 1.006)	<0.0001
	≧288 ng/mL	0.999 (0.997, 1.002)	0.6100
Cancer mortality	<307 ng/mL	1.003 (1.002, 1.004)	<0.0001
	≧307 ng/mL	1.000 (0.998, 1.002)	0.9462
Female	<230 ng/mL	1.006 (1.004, 1.009)	<0.0001
	≧230 ng/mL	1.000 (0.9974 1.003)	0.9217
<65 years	<306 ng/mL	1.003 (1.002, 1.005)	<0.0001
	≧306 ng/mL	1.000 (0.998, 1.002)	0.7718
≧65 years	<278 ng/mL	1.003 (1.001, 1.004)	0.0045
	≧278 ng/mL	0.999 (0.995, 1.003)	0.5266

Meanwhile, threshold saturation effects were observed in the relationships between serum cotinine levels and either all-cause mortality in males (cutoff = 12.1 ng/mL) and females (cutoff = 36.4 ng/mL), cardiovascular disease mortality in males (cutoff = 334 ng/mL), cancer mortality in females (cutoff = 230 ng/mL), cardiovascular disease mortality in younger adults (age < 65 years; cutoff = 288 ng/mL), and cancer mortality in younger (age < 65 years; cutoff = 306 ng/mL), and older adults (age ≥ 65 years cutoff = 278 ng/mL) (Supplementary Figures S2C1–C3, S2B2,B3). Moreover, Serum cotinine levels were associated with a greater risk of all mortality outcomes in females than in males and in the younger adults compared to older adults.

### 3.6 Nomogram for visualizing and validating the multivariate cox regression models

Nomograms were constructed based on the three multivariate Cox regression models. The total points calculated as the sum of the individual points of each of the variables included in the nomogram, and the months of follow-up were 116.5, 115, and 111 months for all-cause, cardiovascular disease, and cancer, respectively (Supplementary Figures S2A1–A3). The AUC of the ROC curve were 0.821 [95% confidence interval (CI) 0.699–0.813], 0.867 (95% CI 0.699–0.813), and 0.833 (95% CI 0.699–0.813) for all-cause, cardiovascular disease, and cancer mortality, respectively (Supplementary Figures S2B1–B3).

## 4 Discussion

This study explored the influence of sex and age on the impact of cigarette exposure on all-cause, cardiovascular disease, and cancer mortality. To the best of our knowledge, no previous study has explored sex-and age-related differences in the association between cigarette exposure and mortality by measuring serum cotinine levels. In summary, our significant findings were that females and younger adults had higher risks of all-cause cardiovascular disease and cancer mortality associated with cigarette exposure. Sex differences were significant only in all-cause and cancer mortality. Threshold saturation effects were observed in all-cause mortality among both males and females, cancer mortality among females, and cardiovascular disease mortality among males. Age differences were significant only in all-cause and cardiovascular disease mortality. Threshold saturation effects were found in cardiovascular disease mortality among younger adults and cancer mortality among all-age population. Furthermore, the nomogram and ROC curves demonstrated the accuracy and reliability of the established models.

Inconsistent with the decreasing trend in overall smoking prevalence worldwide, smoking prevalence in more places changed minimally or increased among women (22). A major finding of this study was that younger adults demonstrated a higher proportion of all-cause, cardiovascular disease, and cancer mortality associated with cigarette exposure than older adults. These age differences are consistent with those reported in previous studies. Hiroyasu et al. demonstrated that the relative risk of smoking-induced cardiovascular disease mortality in Japanese women was greater in persons aged 40–64 years than in older persons (23). Furthermore, many studies have found that early smoking-onset age indicates a high risk of smoking-related mortality. A retrospective study that included 4,499 current or former smokers in a median 7.02 years of follow-up suggested that each year of delay in early smoking-onset age was inversely associated with cardiovascular mortality in the group aged ≤12 years and 4% lower risk of all-cause mortality in all groups (8, 24). However, our study revealed more comprehensive findings that the relationship between serum cotinine and cardiovascular disease in younger adults, and cancer mortality in all-age population showed threshold saturation effects. Additionally, the positive correlation was only significant in cancer mortality among older adults. For cardiovascular diseases mortality, the risk in younger adults was increased with increasing serum cotinine, while when serum cotinine increased to 306 ng/mL, this dose–response trend was no longer significant. There were, however, no dose–response trends in older adults. For cancer mortality, the risk was increased with increasing serum cotinine in all ages and the increased risk was early weakened in older adults after serum cotinine increased beyond the threshold, compared to younger adults. This implies that there is a dose-effect relationship in smoking-related carcinogenesis among all ages, while smoking-related atherosclerosis does not present a dose-effect relationship in older adults. Our results suggested that a delay in early smoking-onset age reduced the risk of smoking-induced cardiovascular disease mortality but did not significantly decrease the risk of smoking-induced cancer mortality.

Females have greater health consequences associated with tobacco exposure. For example, earlier large-scale cohort studies in the Asia-Pacific region reported that women presented a two-fold higher risk of lung cancer mortality associated with current smoking in Australia and New Zealand than men (2). Similarly, increased all-cause mortality in females from myocardial infarction was previously reported (5). Hurley found that for women, the all-cause mortality risk induced by light smoking may be approximately three times that for men, whereas no such relationship was observed for medium/heavy smoking (9), which was consistent with our study. We found that females had a higher risk of all-cause and cancer mortality than males before serum cotinine increased to the threshold. However, for cardiovascular diseases mortality, the risk was increased simultaneously with increasing serum cotinine in males and females and the increased risk was early weakened in males after serum cotinine rose beyond the threshold. This indicates that mild and moderate tobacco exposure resulted in a higher risk of all-cause and cancer mortality in females and heavy tobacco exposure brought little risk of mortality in males and females. Interestingly, the harm of cardiovascular diseases from mild and moderate tobacco exposure in females and males is approximately equal.

One of the underlying mechanisms of the sex difference may be that due to the anti-estrogenic effect of smoking (25). Women smokers suffer from the twice “struck” phenomena: smoking directly increases the risk of ischemic heart disease by inducing atherosclerosis in both women and men, and in women, smoking may substantially attenuate the protective role of estrogen in atherosclerosis, and gastric and colon cancers (26–28). Another underlying mechanism for their higher risk of all-cause mortality is because women are more vulnerable to chronic obstructive pulmonary disease (29). Additionally, smoking cessation is more difficult in females than males (30, 31). Females experience more weight gain related to smoking cessation worries and suffer severe side effects from smoking cessation medications and craving, leading to fewer antismoking medications (32–35). Cepeda-Benito et al. reported that nicotine replacement therapy is less effective in females than males (35). Difficulty in quitting smoking increases the lasting detrimental effects of smoking in women who attempt to quit.

The age difference effect is relatively easy to understand. A prospective study reported that younger smokers have a greater mortality risk from cancer than those who start later (7). Similar results have been reported for coronary heart disease (36). The initiation of premature smoking is typically associated with more difficult cessation. This study suggests that younger adults are at a higher risk of smoking-related mortality. To interpreting this result, possibility of a cumulative effect need be considered; younger adults with the same serum cotinine level as the older adults may have a longer duration of smoking exposure (37).

In the present study, dose–response relationships and saturation effects of tobacco exposure showed sex and age differences, while sex differences in cardiovascular disease and age differences in cancer mortality were not significant; these results were not found in the previous study. These results suggest that and sex and age differences and corresponding saturation effects in smoking-related mortality are inconsistent in different diseases. Finally, this study included several laboratory parameters and demographic data. Most underlying diseases from the more representative and multiracial samples of the US population were excluded, which improved the reliability and accuracy of the study. The major study limitations were the long-term prognostic outcome, and a single point detection of serum cotinine concentration may not completely reflect the degree of smoking exposure during the follow-up period.

## 5 Conclusion

Taken together, our study demonstrated sex and age differences in smoking risk by measuring serum cotinine levels. The findings of dose–response relationships and threshold saturation effects provide evidence that mild and moderate tobacco exposure brought a higher risk of all-cause and cancer mortality in females. It has been noted that sex differences in cardiovascular disease and age differences in cancer mortality were insignificant, indicating inconsistency in different diseases. Cotinine could be an effective indicator to reveal novel findings for sex and age differences in smoking risk.

## Data Availability

The original contributions presented in the study are included in the article/Supplementary material, further inquiries can be directed to the corresponding authors.
