# Supplementary material for: Beyond self-reports: serum cotinine reveals sex-and age-related differences of smoking on all-cause and disease-specific mortality
Source: Front Public Health. 2025 Feb 17;13:1512603. doi: 10.3389/fpubh.2025.1512603 (PMC11873280; doi:10.3389/fpubh.2025.1512603)

**Supplementary Material**

# **Supplementary methods**

Variables

Routine biochemistry profiles and complete blood count (CBC)

For the CBC parameters, the combination of an automatic diluting and mixing device was used for sample processing, and a single-beam photometer was used to count and assess for hemoglobinopathy. White blood cell (WBC) differential analysis was performed using the (volume, conductivity, and scatter (VCS) technology. Bichromatic digital endpoint method was used to determine the serum albumin concentration in all NHANES cycles. In the NHANES 2005-2007 and 2008-2010/2015-2018 alanine aminotransferase (ALT), aspartate aminotransferase (AST), and alkaline phosphatase (ALP) levels were analyzed using enzymatic and kinetic rate methods, respectively. In all NHANES cycles, gamma-glutamyl transferase (GGT) and serum creatinine levels were analyzed using the enzymatic and Jaffe (calibrated and traceable to an isotope-dilution mass spectrometry reference method) rate methods, respectively. Standard creatinine values (mg/dL) were corrected using the equation: -0.016 + 0978* (NHANES 05–06 uncalibrated serum creatinine, mg/dL).

Blood urea nitrogen (BUN)

BUN was analyzed using the enzymatic conductivity rate method, and the concentrations of sodium, calcium, chloride, bicarbonate, and potassium in the serum were measured using indirect ion selective electrode (ISE) methodology. Corrected calcium levels were calculated by adjusting for serum albumin levels when the albumin levels were <34 g/L. The LX20 and DxC800 systems used a timed-rate method to determine the concentration of phosphorus in the serum, and the oxygen rate method was used to measure serum glucose concentrations. Total triglyceride, uric acid, iron, and total cholesterol levels were analyzed using the timed-endpoint method. The DxC800 and LX20 systems used a timed-endpoint diazo method (Jendrassik–Grof) to measure the total serum bilirubin concentration. The total protein concentration was analyzed using the time-rate biuret method, whereas globulin was calculated by subtracting albumin from the total protein. The LX20 and DxC800 systems used a lactate dehydrogenase (LDH) reagent based on an enzymatic rate method to measure serum LDH activity in all NHANES cycles.

# **Supplementary Figures and Tables**

# **Supplementary Tables**

**Supplementary table S1.** Baseline characteristics of participants, NHANES 1999-2018 (N=16393)

| **Variables** | **Males**  **(n=8678)** | **Females (n=7715)** | ***P*** |
| --- | --- | --- | --- |
| **Standard Biochemistry Profile** |  |  |  |
| Alanine Aminotransferase (ALT) (U/L) | 28.62 ± 18.73 | 20.56 ± 13.56 | <0.001 |
| Albumin, serum (g/L) | 43.52 ± 3.15 | 41.38 ± 3.57 | <0.001 |
| Alkaline Phosphatase (ALP) (IU/L) | 69.40 ± 21.15 | 68.73 ± 23.87 | <0.001 |
| Aspartate Aminotransferase (AST) (U/L) | 26.53 ± 16.54 | 22.53 ± 11.24 | <0.001 |
| Bicarbonate (mmol/L) | 25.26 ± 2.21 | 24.53 ± 2.32 | <0.001 |
| Blood Urea Nitrogen (BUN) (mg/dL) | 4.90 ± 1.76 | 4.33 ± 1.78 | <0.001 |
| Chloride (mmol/L) | 103.20 ± 2.87 | 103.72 ± 2.92 | <0.001 |
| Globulin (g/L) | 28.49 ± 4.30 | 29.50 ± 4.41 | <0.001 |
| Serum glucose (mmol/L) | 5.65 ± 2.02 | 5.41 ± 1.81 | <0.001 |
| Gamma Glutamyl Transferase (GGT) (U/L) | 32.82 ± 44.20 | 23.11 ± 35.43 | <0.001 |
| Iron, refrigerated serum (umol/L) | 16.80 ± 6.24 | 14.11 ± 6.21 | <0.001 |
| Lactate Dehydrogenase (LDH) (U/L) | 131.45 ± 31.66 | 130.25 ± 28.50 | <0.001 |
| Osmolality (mmol/Kg) | 278.84 ± 4.84 | 277.39 ± 5.14 | <0.001 |
| Phosphorus (mg/dL) | 1.18 ± 0.18 | 1.23 ± 0.17 | <0.001 |
| Potassium (mmol/L) | 4.03 ± 0.33 | 3.93 ± 0.33 | <0.001 |
| Sodium (mmol/L) | 139.45 ± 2.29 | 139.11 ± 2.33 | <0.001 |
| Total Bilirubin (umol/L) | 12.28 ± 5.43 | 9.55 ± 4.39 | <0.001 |
| Total Calcium (mmol/L) | 2.36 ± 0.09 | 2.34 ± 0.09 | <0.001 |
| Cholesterol, refrigerated serum (mmol/L) | 5.04 ± 1.07 | 5.12 ± 1.07 | <0.001 |
| Total Protein (g/L) | 72.00 ± 4.51 | 70.86 ± 4.68 | <0.001 |
| Uric acid (umol/L) | 357.66 ± 75.01 | 281.78 ± 72.15 | <0.001 |
| **Complete Blood Count - Whole Blood** |  |  |  |
| White blood cell count (1000 cells/uL) | 7.30 ± 2.57 | 7.57 ± 5.02 | <0.001 |
| Lymphocyte number (1000 cells/uL) | 2.17 ± 1.40 | 2.32 ± 4.15 | <0.001 |
| Monocyte number (1000 cells/μL) | 0.59 ± 0.21 | 0.54 ± 0.18 | <0.001 |
| Neutrophils number (1000 cell/uL) | 4.28 ± 1.82 | 4.48 ± 1.85 | <0.001 |
| Eosinophils number (1000 cells/uL) | 0.22 ± 0.19 | 0.19 ± 0.15 | <0.001 |
| Basophils number (1000 cells/uL) | 0.05 ± 0.06 | 0.05 ± 0.06 | <0.001 |
| Red blood cell count (million cells/uL) | 4.96 ± 0.45 | 4.48 ± 0.39 | <0.001 |
| Hemoglobin (g/dL) | 15.11 ± 1.19 | 13.39 ± 1.21 | <0.001 |
| Hematocrit (%) | 44.46 ± 3.34 | 39.72 ± 3.33 | <0.001 |
| Mean cell volume (MCV) (fL) | 89.90 ± 5.13 | 88.91 ± 6.00 | <0.001 |
| Mean cell hemoglobin (pg) | 30.54 ± 2.07 | 29.99 ± 2.50 | <0.001 |
| Mean cell hemoglobin concentration (g/dL) | 33.97 ± 0.94 | 33.70 ± 1.02 | <0.001 |
| Red cell distribution width (RDW) (%) | 13.08 ± 0.94 | 13.37 ± 1.35 | <0.001 |
| Platelet count (1000 cells/uL) | 236.74 ± 57.64 | 264.66 ± 65.36 | <0.001 |
| Mean platelet volume (fL) | 8.17 ± 0.90 | 8.25 ± 0.92 | <0.001 |
| **Total Nutrient Intakes** |  |  |  |
| Total energy intake (kcal/day) | 2528.35 ± 1151.67 | 1879.03 ± 817.03 | <0.001 |
| Total protein intake (gm/day) | 97.83 ± 50.44 | 70.58 ± 33.83 | <0.001 |
| Total carbohydrate intake (gm/day) | 295.64 ± 146.43 | 228.39 ± 108.44 | <0.001 |
| Total sugars intake (gm/day) | 129.02 ± 93.46 | 104.11 ± 70.13 | <0.001 |
| Total dietary fiber intake (gm/day) | 18.36 ± 11.57 | 15.06 ± 9.27 | <0.001 |
| Total fat intake (gm/day) | 95.97 ± 54.67 | 73.26 ± 40.66 | <0.001 |
| Total saturated fatty acids intake (gm/day) | 31.23 ± 19.74 | 23.66 ± 14.49 | <0.001 |
| Total monounsaturated fatty acids intake (gm/day) | 34.80 ± 20.78 | 25.92 ± 15.31 | <0.001 |
| Total polyunsaturated fatty acids intake (gm/day) | 21.18 ± 14.20 | 17.08 ± 11.45 | <0.001 |
| Total cholesterol intake (mg/day) | 362.12 ± 286.11 | 258.20 ± 208.53 | <0.001 |
| Vitamin B1 intake (mg/day) | 1.85 ± 1.05 | 1.38 ± 0.74 | <0.001 |
| Vitamin B2 intake (mg/day) | 2.45 ± 1.61 | 1.84 ± 1.01 | <0.001 |
| Niacin intake (mg/day) | 30.73 ± 18.78 | 21.19 ± 11.39 | <0.001 |
| Vitamin B6 intake (mg/day) | 2.47 ± 1.99 | 1.72 ± 1.16 | <0.001 |
| Total folate intake (mcg/day) | 449.24 ± 279.19 | 347.71 ± 207.65 | <0.001 |
| Added vitamin B12 intake (mcg/day) | 6.10 ± 7.61 | 4.08 ± 4.15 | <0.001 |
| Vitamin C intake (mg/day) | 89.02 ± 109.63 | 77.33 ± 87.55 | <0.001 |
| Calcium intake (mg/day) | 1030.87 ± 695.30 | 839.28 ± 503.62 | <0.001 |
| Phosphorus intake (mg/day) | 1583.26 ± 797.06 | 1189.34 ± 559.46 | <0.001 |
| Magnesium intake (mg/day) | 335.10 ± 166.26 | 265.58 ± 133.37 | <0.001 |
| Magnesium intake (mg/day) | 16.76 ± 9.48 | 12.71 ± 7.03 | <0.001 |
| Zinc intake (mg/day) | 13.59 ± 11.01 | 9.61 ± 5.42 | <0.001 |
| Copper intake (mg/day) | 1.41 ± 1.16 | 1.13 ± 0.74 | <0.001 |
| Sodium intake (mg/day) | 4089.55 ± 2113.42 | 3062.70 ± 1529.45 | <0.001 |
| Potassium intake (mg/day) | 2988.27 ± 1418.81 | 2341.69 ± 1088.20 | <0.001 |
| Selenium intake (mcg/day) | 134.84 ± 76.81 | 98.00 ± 54.38 | <0.001 |
| Caffeine intake (mg/day) | 185.22 ± 251.38 | 149.90 ± 189.98 | <0.001 |
| Theobromine intake (mg/day) | 36.35 ± 84.94 | 37.29 ± 79.66 | <0.001 |
| Alcohol intake (mg/day) | 16.85 ± 36.26 | 7.10 ± 19.98 | <0.001 |
| Glycohemoglobin (%) | 5.70 ± 1.02 | 5.60 ± 0.95 | <0.001 |
| Direct HDL-Cholesterol (mmol/L) | 1.25 ± 0.37 | 1.51 ± 0.43 | <0.001 |
| Estimated glomerular filtration rate (eGFR) (ml/min) | 119.91 ± 44.00 | 119.45 ± 49.55 | <0.001 |

# **Supplementary Figure1**


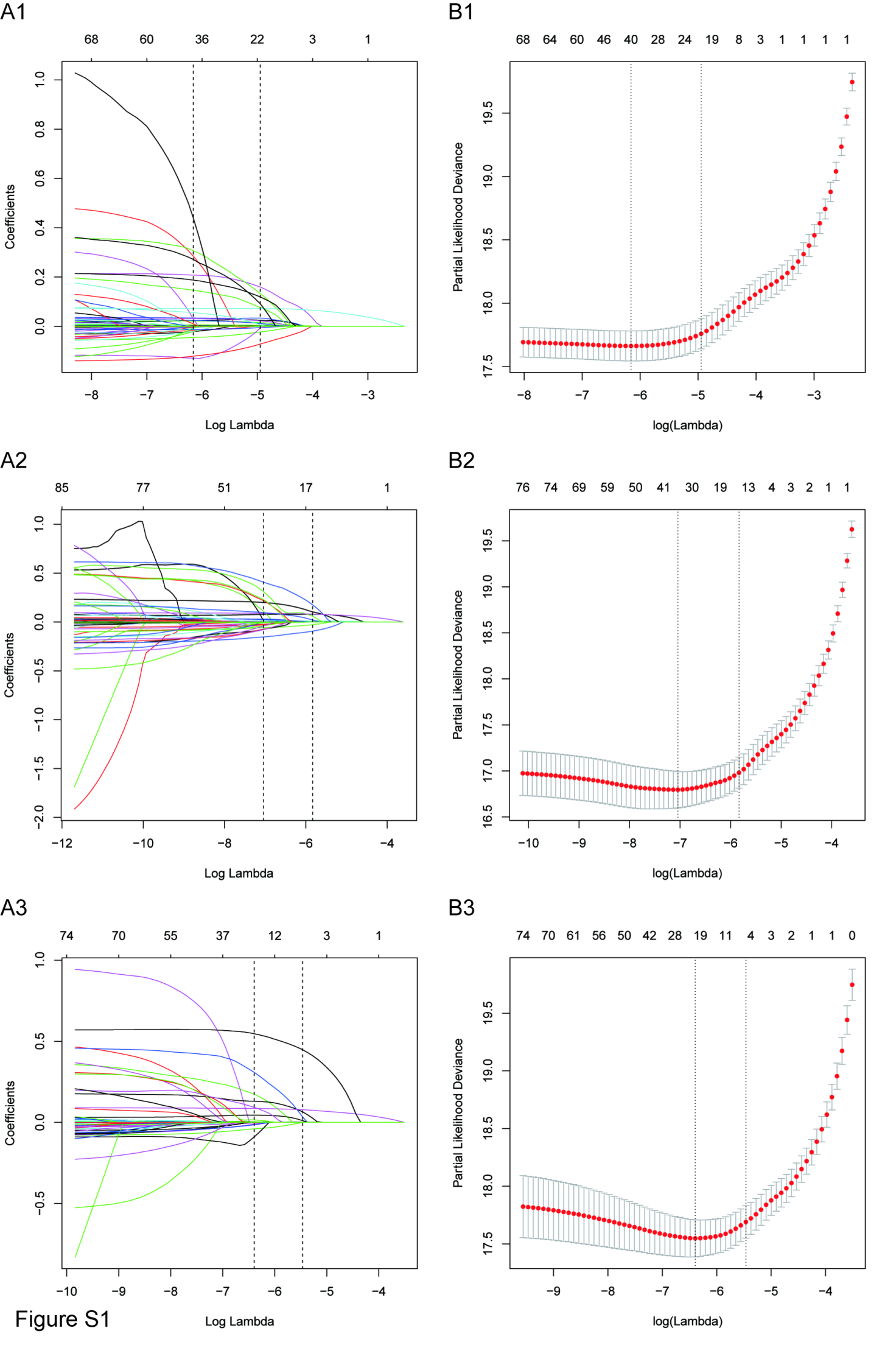

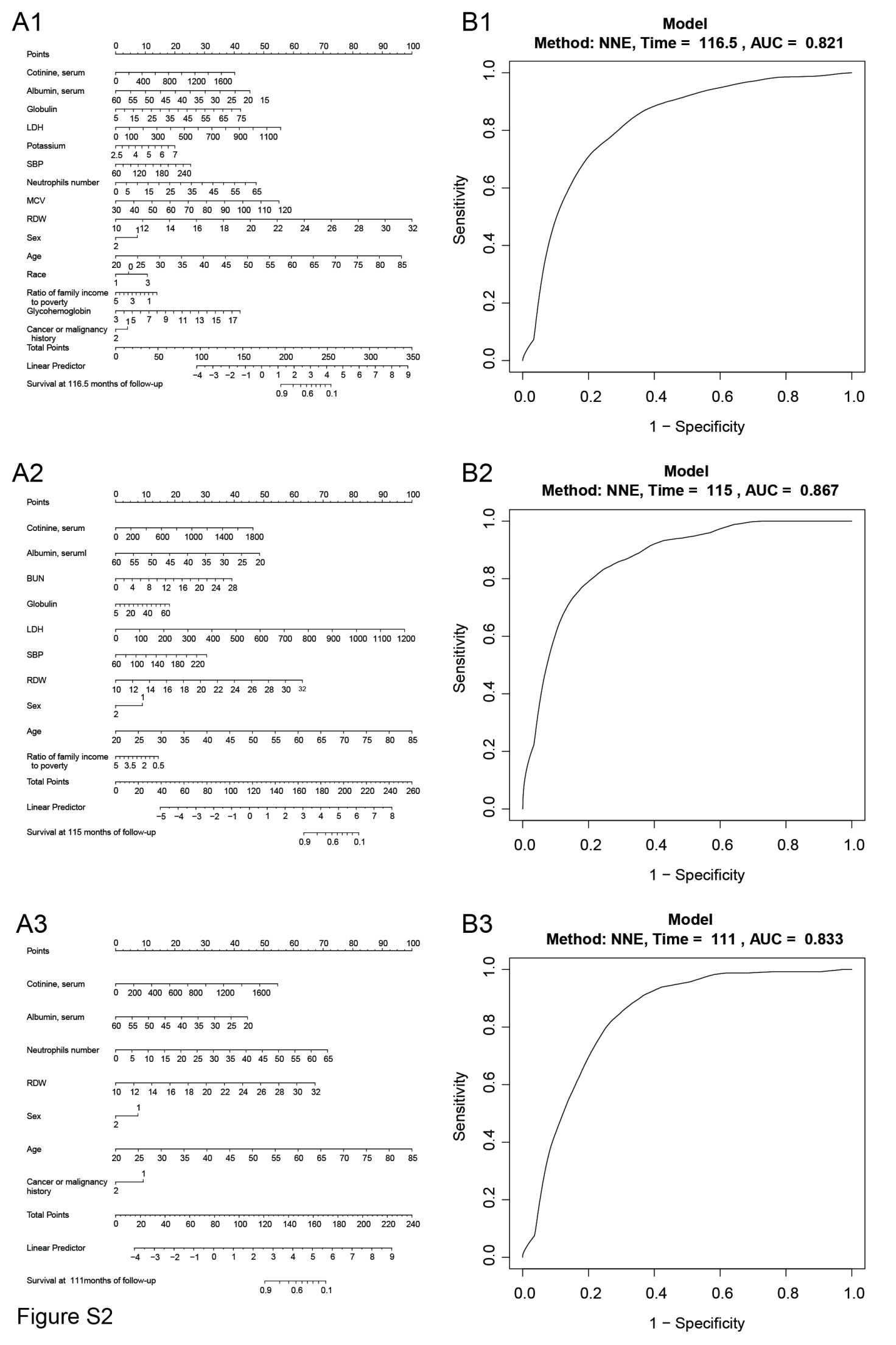

Supplement: Supplementary file 1 [file Data_Sheet_1.docx]
